# Real-World Evaluation of a Trastuzumab Emtansine Biosimilar (Ujvira®) in Human Epidermal Growth Factor Receptor 2 (HER2)-Positive Metastatic Breast Cancer

**DOI:** 10.7759/cureus.103700

**Published:** 2026-02-16

**Authors:** MV Chandrakanth, Vivek Agarwala, Rupam Manna, Amit Sharma, Minakshi Roy, Anish Dasgupta, Kaustav Mandal, Nibedita Sen, Pritam K Sardar, Aparajita Sadhya, Moinak Basu, Subhabrata Kumar, Himadri Nayak, Vipulkumar Thummar, Priya Mehta

**Affiliations:** 1 Medical Oncology, Narayana Superspeciality Hospital, Kolkata, IND; 2 Medical Oncology, Rabindranath Tagore International Institute of Cardiac Sciences, Kolkata, IND; 3 Medical Affairs, Zydus Lifesciences Ltd, Ahmedabad, IND

**Keywords:** her2-positive breast cancer, metastatic breast cancer, progression-free survival, real-world evidence, trastuzumab emtansine biosimilar, ujvira®

## Abstract

Background: Trastuzumab emtansine (T-DM1) is widely regarded as a standard treatment for human epidermal growth factor receptor 2 (HER2)-positive metastatic breast cancer (MBC), but its cost puts it beyond the reach of many patients in low- and middle-income countries. Ujvira® (ZRC-3256; Zydus Lifesciences, India), a biosimilar version, could offer a more affordable alternative, although real-world experience with it is still limited.

Methods: This retrospective, single-center study evaluated the efficacy and safety of Ujvira® in patients with HER2-positive MBC treated at a tertiary cancer center in India. All patients had prior trastuzumab exposure and received Ujvira® 3.6 mg/kg intravenously every 21 days until progression or unacceptable toxicity. The primary endpoint was progression-free survival (PFS), estimated by the Kaplan-Meier method. Secondary endpoints included objective response rate (ORR), safety (graded per CTCAE v5.0), and exploratory subgroup analyses based on ECOG performance status, hormone receptor expression, comorbidities, and metastatic pattern.

Results: Fifty-one patients were included (mean age 58.0 ± 8.4 years; 47 (92.2%) postmenopausal). Median PFS was 6.9 months (95% CI 6.2-9.0) overall, 7.7 months in the second-line setting, and 9.1 months in the third-line setting. The ORR was 35.3%. Treatment was well tolerated; the most common adverse events were thrombocytopenia in 17 patients (33.3%) and anemia in 10 patients (19.6%). Dose reductions occurred in eight patients (15.7%), which did not affect efficacy (PFS 6.8 vs 6.9 months; p = 0.25).

Conclusions: In this real-world Indian cohort, Ujvira® demonstrated efficacy and safety consistent with innovator T-DM1, with preserved outcomes even among heavily pretreated and comorbid patients. These results support Ujvira® as an effective, tolerable, and accessible therapeutic option for HER2-positive MBC in resource-limited settings.

## Introduction

Human epidermal growth factor receptor 2 (HER2)-positive breast cancer accounts for approximately 20-25% of all breast cancers and is associated with aggressive biology, higher recurrence rates, and poorer outcomes compared with HER2-negative disease [1]. The advent of HER2-directed therapies has transformed management, markedly improving survival. Trastuzumab remains the backbone of therapy, and the antibody-drug conjugate trastuzumab emtansine (T-DM1) is established as the standard second-line option, based on pivotal phase III trials such as EMILIA and TH3RESA, which demonstrated significant improvements in progression-free survival (PFS) and overall survival (OS) compared with the physician’s choice of treatment [2,3].

Although this has proven effective, high cost and limited access in low- and middle-income countries (LMICs) restrict the widespread use of innovator T-DM1 (Kadcyla®) in these settings [4]. Biosimilars offer a lower-priced option that expands access and has similar efficacy and safety. Ujvira® (ZRC-3256; Zydus Lifesciences, India) is a trastuzumab emtansine biosimilar that received approval after a randomized, open-label, multicenter comparative trial showed analytical, pharmacokinetic, efficacy, and immunogenicity similarities to the original product [5]. Systematic reviews, including Cochrane analyses, also confirm clinical equivalence of monoclonal antibody biosimilars in oncology [6].

There is limited published real-world information on trastuzumab emtansine biosimilars. Case reports have reported long-term responses in patients with brain metastases [7]. The majority of existing evidence is, however, limited to small cohorts or anecdotal experience, and very little has been done to understand comorbidities, biomarker subgroups, or the outcome of dose alterations.

The current paper fills this gap by assessing the effectiveness and safety of Ujvira® in a single-center real-world cohort of 51 patients with HER2-positive metastatic breast cancer. To our knowledge, it is the first real-world study to examine PFS in various clinically relevant subgroups, such as brain metastases, hormone receptor, FISH status, endocrine therapy, comorbidity burden (diabetes, hypertension), menopausal status, and dose reduction. This review aims to provide practical information about the use of trastuzumab emtansine biosimilars in clinical oncology practice, specifically in resource-limited settings.

## Materials and methods

Study design and setting

This was a retrospective, single-center, observational study carried out in a tertiary oncology hospital in India. The researchers analyzed the effectiveness and safety of the trastuzumab emtansine biosimilar Ujvira® (Zydus Lifesciences) in metastatic patients with HER2-positive breast cancer. The Institutional Review Board approved the study. Since the data collection was retrospective in nature, the requirement for individual informed consent was waived. This research was conducted in accordance with the principles of the Declaration of Helsinki and national regulatory guidelines.

Participants

Eligible patients included those diagnosed with HER2-positive metastatic breast cancer who received Ujvira® between July 2022 and December 2024. All patients had previously been treated with trastuzumab and had undergone at least one course of chemotherapy, either in the adjuvant or metastatic setting. Patients were eligible if they had sufficient clinical, treatment, and follow-up data. The study group had a high incidence of comorbidities, such as diabetes mellitus and hypertension, and prior exposure to various systemic treatments, including taxanes, anthracyclines, pertuzumab, and lapatinib. Baseline data were documented, including Eastern Cooperative Oncology Group (ECOG) performance status, hormone receptor status (ER and PR), fluorescence in situ hybridization (FISH) result, and HER2 immunohistochemistry (IHC) score, and metastatic pattern (visceral, non-visceral, and brain involvement) [8,9].

Treatment protocol

All patients received Ujvira® intravenously at a dose of 3.6 mg/kg every 21 days until disease progression, unacceptable toxicity, or treatment withdrawal. Dose modifications were permitted in the event of treatment-related adverse events. Patients requiring dose reductions were analyzed as a distinct subgroup.

Outcomes

The primary endpoint was PFS, defined as the time from initiation of Ujvira® to documented disease progression or death from any cause. Secondary endpoints included objective response rate (ORR), assessed radiologically using RECIST version 1.1; safety outcomes, including incidence and severity of treatment-related adverse events; and exploratory subgroup analyses of PFS conducted over the entire follow-up period from initiation of Ujvira® until disease progression or censoring, stratified by brain metastases, line of therapy, endocrine therapy status, ECOG score, menopausal status, hormone receptor expression, HER2 IHC score, FISH status, comorbidities (diabetes mellitus, hypertension), visceral versus non-visceral involvement, radiotherapy, and dose reduction status [10].

Safety monitoring included clinical assessment at each treatment visit. Laboratory investigations, including complete blood count, liver function tests, renal function tests, and serum electrolytes, were performed at baseline and prior to each treatment cycle. Severity of treatment-related adverse events was graded per the Common Terminology Criteria for Adverse Events (CTCAE) version 5.0.

Imaging and response assessment were performed using contrast-enhanced CT (CECT) of the chest, abdomen, and pelvis at baseline and every 8-12 weeks, or earlier if clinically indicated. Tumor response was assessed according to RECIST v1.1 criteria by institutional radiologists (Appendices).

Statistical analysis

Descriptive statistics were used to summarize baseline demographic and clinical characteristics. PFS was estimated using the Kaplan-Meier method, and median survival was reported with 95% confidence intervals (CIs). Subgroup comparisons were conducted using the log-rank test. All analyses were performed using IBM SPSS Statistics for Windows, Version 25 (Released 2017; IBM Corp., Armonk, New York). A two-sided p-value < 0.05 was considered statistically significant.

## Results

Baseline characteristics

A total of 51 female patients with HER2-positive metastatic breast cancer were included; 40 (78.4%) were receiving second-line therapy, while 11 (21.6%) were on third-line therapy. The mean age was 58 years (SD 8.4), and almost half (49.0%) were aged ≥60 years. The majority of the patients were postmenopausal, 47 (92.2%), and the most common ECOG performance status was 1 (64.7%). There was also a high prevalence of comorbidities, with hypertension in 31 (60.8%) patients and diabetes mellitus in 30 (58.8%) patients. The disease burden was high, with more than 40% of patients having ≥3 metastatic sites at baseline.

Most tumors were hormone receptor-positive (ER: 70.6%; PR: 54.9%). HER2 IHC 3+ was positive in 38 patients (74.5%), and 12 patients (23.5%) were FISH-positive. All patients were previously exposed to trastuzumab, and most were heavily pretreated with taxanes, 43 (84.3%), and anthracyclines, 32 (62.7%). Exposure to other HER2-directed agents was also significant: lapatinib in 20 (39.2%) and pertuzumab in 10 (19.6%) patients. Capecitabine was previously used in 17 (33.3%) patients (Table 1).

**Table 1:** Baseline demographic and clinical characteristics of patients treated with Ujvira® (ZRC-3256; Zydus Lifesciences, India) (N=51) SD: standard deviation, ECOG: Eastern Cooperative Oncology Group performance status, ER: oestrogen receptor, PR: progesterone receptor, HER2: human epidermal growth factor receptor 2, IHC: immunohistochemistry, FISH: fluorescence in situ hybridisation.

Parameter	Values
Age, mean ± SD (years)	58.0 ± 8.4
<60 years, n (%)	26 (51.0)
≥60 years, n (%)	25 (49.0)
Postmenopausal, n (%)	47 (92.2)
Premenopausal, n (%)	4 (7.8)
Hypertension, n (%)	31 (60.8)
Diabetes mellitus, n (%)	30 (58.8)
Hypothyroidism, n (%)	5 (9.8)
ECOG 0, n (%)	18 (35.3)
ECOG 1, n (%)	33 (64.7)
ER positive, n (%)	36 (70.6)
PR positive, n (%)	28 (54.9)
HER2 IHC 3+, n (%)	38 (74.5)
FISH positive, n (%)	12 (23.5)
Prior trastuzumab, n (%)	51 (100.0)
Prior taxane, n (%)	43 (84.3)
Prior anthracycline, n (%)	32 (62.7)
Prior lapatinib, n (%)	20 (39.2)
Prior capecitabine, n (%)	17 (33.3)
Prior pertuzumab, n (%)	10 (19.6)

Progression-free survival

**Figure 1:**
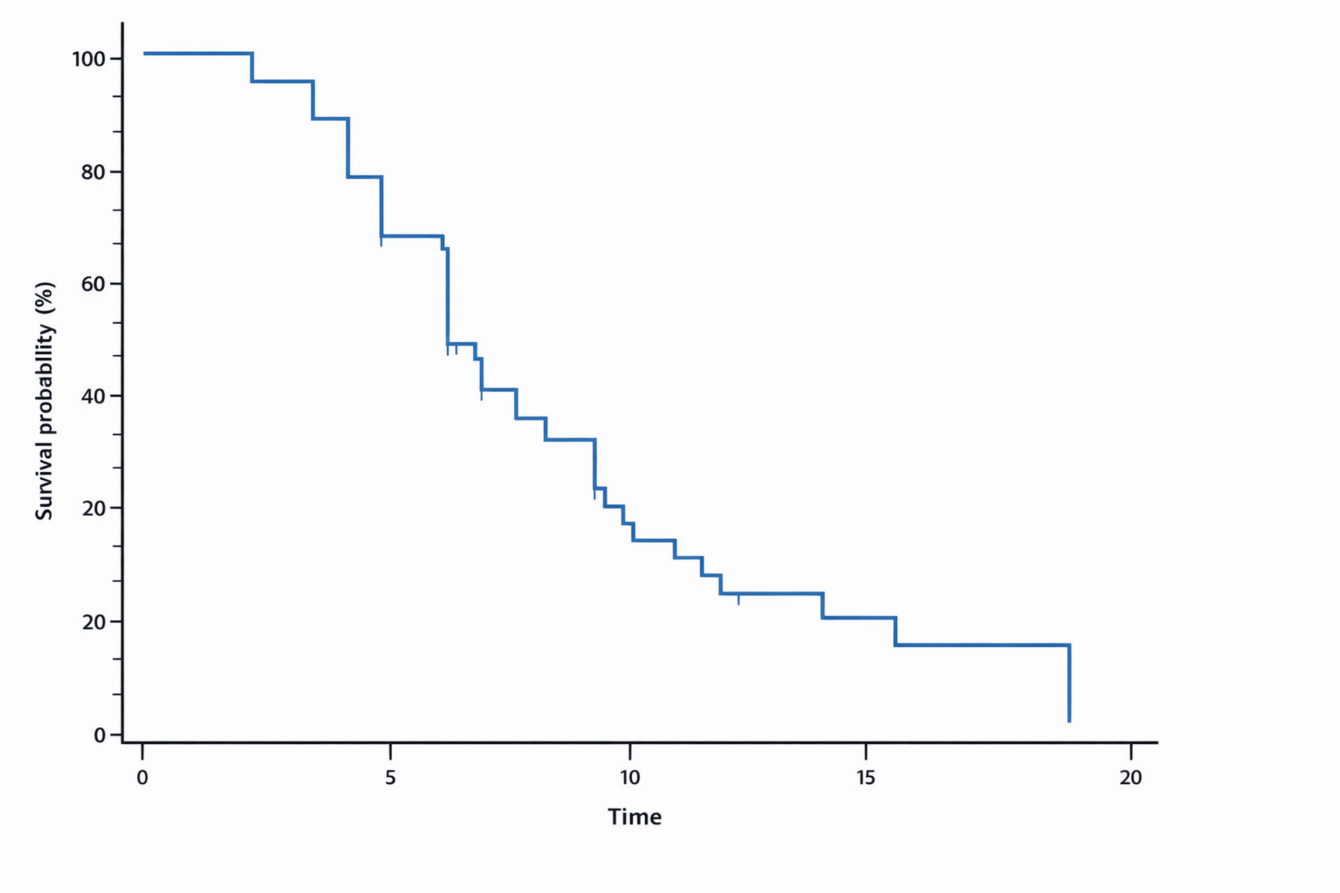
Kaplan–Meier survival curve for overall progression-free survival (PFS) in the study cohort (N=51)

Median PFS for the overall cohort was 6.9 months (95% CI: 6.2-9.0) (Figure 1). Patients treated with Ujvira® in the second-line setting had a median PFS of 7.7 months (95% CI: 6.2-10.5), while those treated in the third-line setting had a median PFS of 9.1 months (95% CI: 1.47-undefined). The ORR was 35.3% (Figure 1).

Subgroup analyses

Brain Metastases

**Figure 2:**
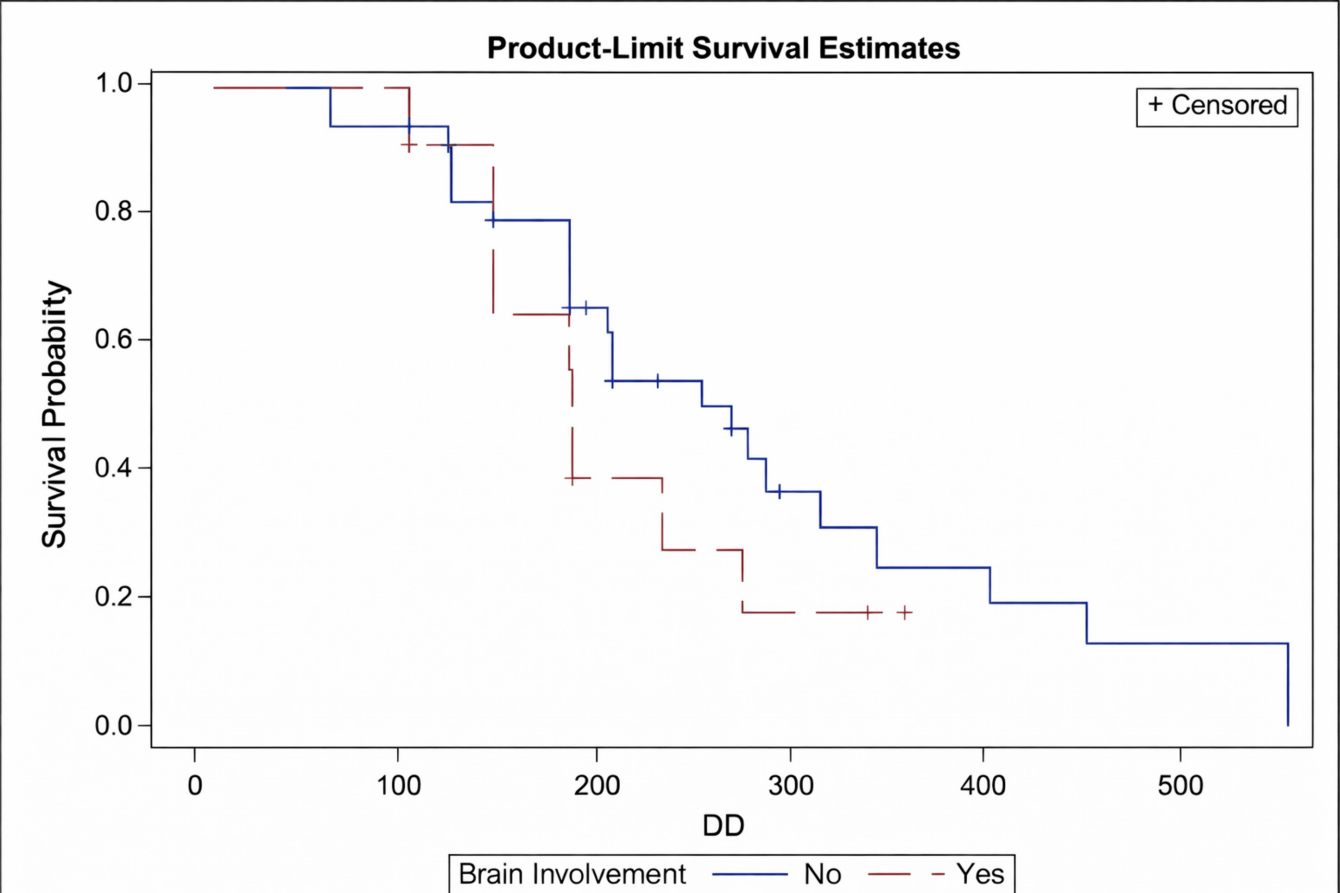
Kaplan–Meier survival curve comparing PFS between patients with (red) and without (blue) brain involvement

Patients with brain metastases had a median PFS of 6.3 months (95% CI: 147-273 days) versus 9.1 months (95% CI: 208-351 days) in those without brain metastases. The difference was not statistically significant (p = 0.2203), although the trend suggests poorer outcomes with CNS involvement (Figure 2).

PFS by Clinical and Biomarker Subgroups

Exploratory subgroup analyses demonstrated numerical differences in median PFS across clinical and biomarker-defined subgroups, with no statistically significant differences observed. Patients with diabetes mellitus had a median PFS of 7.0 months compared to 9.37 months in non-diabetic patients (p = 0.2405). PR-positive tumors showed 9.37 months versus 7.7 months in PR-negative cases (p = 0.3409), while ER-positive patients had 7.7 months compared to 9.1 months in ER-negative patients (p = 0.8705). FISH-positive cases had 9.77 months versus 7.0 months in those in whom testing was not applicable (p = 0.216), and HER2 IHC 2+ tumors had 9.77 months compared with 7.0 months in 3+ tumors (p = 0.3148).

Median PFS was 9.37 months in ECOG 0 versus 8.33 months in ECOG 1 (p = 0.1797), 9.1 months in premenopausal versus 7.0 months in postmenopausal women (p = 0.5551), and 9.1 months in hypertensive versus 7.7 months in non-hypertensive patients (p = 0.3735). Patients receiving radiotherapy for brain metastases had shorter PFS (6.23 vs 9.1 months, p = 0.1157) (Table 2).

**Table 2:** Median PFS across clinical and biomarker subgroups PFS: progression-free survival, PR: progesterone receptor, ER: oestrogen receptor, FISH: fluorescence in situ hybridisation, HER2: human epidermal growth factor receptor 2, IHC: immunohistochemistry, ECOG: Eastern Cooperative Oncology Group performance status.

Subgroup	Comparison groups	Median PFS (months)
Diabetes mellitus	With vs without	7.0 vs 9.37
PR status	Positive vs negative	9.37 vs 7.7
ER status	Positive vs negative	7.7 vs 9.1
FISH status	Positive vs not applicable	9.77 vs 7.0
HER2 IHC score	2+ vs 3+	9.77 vs 7.0
ECOG performance	0 vs 1	9.37 vs 8.33
Menopausal status	Premenopausal vs postmenopausal	9.1 vs 7.0
Hypertension	With vs without	9.1 vs 7.7
Visceral involvement	Present vs absent	8.33 vs 7.0
Non-visceral involvement	Present vs absent	9.1 vs 8.33
Radiotherapy for brain metastases	Yes vs no	6.23 vs 9.1

Line of therapy

**Figure 3:**
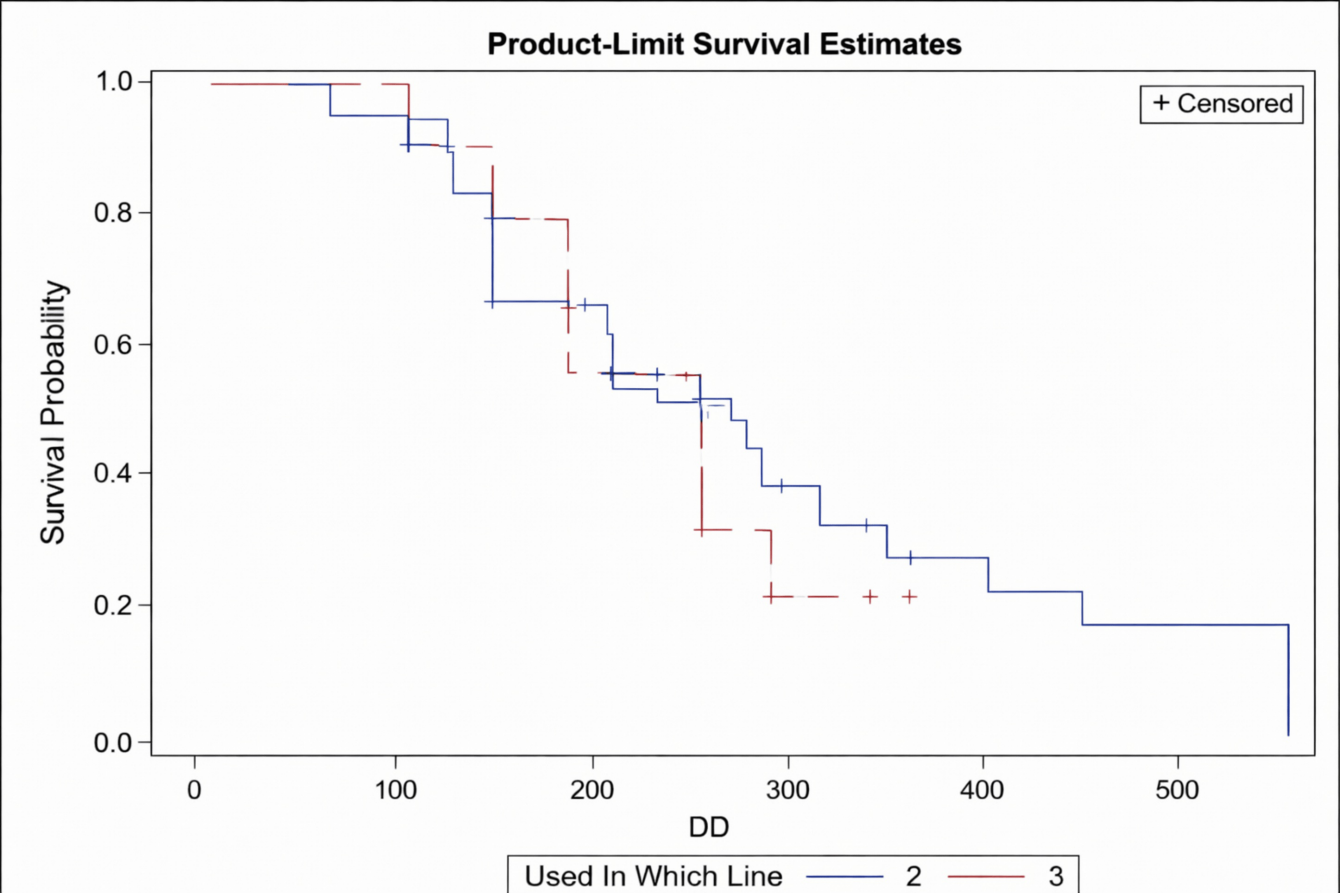
Kaplan–Meier survival curve showing PFS for patients receiving the drug in second-line (blue) versus third-line (red) settings

Median PFS was 7.7 months (95% CI: 189-321 days) in patients treated in the second-line setting and 9.1 months (95% CI: 147-undefined) in those treated in the third-line setting. The difference was not statistically significant (p = 0.9335) (Figure 3).

Safety and dose modifications

Treatment with Ujvira® was generally well tolerated. The most common adverse events were thrombocytopenia in 17 (33.3%), anemia in 10 (19.6%), and neutropenia in 4 patients (7.8%). Elevated transaminases occurred in 2 (3.9%), hyperglycemia in 3 (5.9%), and reduced left ventricular ejection fraction (LVEF <50%) in 2 patients (3.9%). Most events were manageable with supportive care (Table 3).

**Table 3:** Treatment-related adverse events in patients receiving Ujvira® (N=51) Adverse events were graded according to the Common Terminology Criteria for Adverse Events (CTCAE), version 5.0. Grade ≥3 toxicities were infrequent; detailed grading was available only for elevated transaminases.

Adverse event	Any grade, n (%)	Grade ≥3, n (%)
Thrombocytopenia	17 (33.3)	Not reported
Anemia	10 (19.6)	Not reported
Neutropenia	4 (7.8)	Not reported
Elevated transaminases	2 (3.9)	2 (3.9)
Hyperglycemia	3 (5.9)	Not reported
Left ventricular ejection fraction <50%	2 (3.9)	Not reported

Dose reductions were required in eight patients (15.7%), all due to treatment-related toxicities. Importantly, efficacy was preserved despite dose modification, with a median PFS of 6.8 months (95% CI: 4.1-18.6) in patients with dose reductions versus 6.9 months (95% CI: 6.2-9.0) in those without (p = 0.25).

## Discussion

In this retrospective real-world analysis of 51 Indian patients with HER2-positive metastatic breast cancer treated with the trastuzumab emtansine biosimilar Ujvira®, we observed a median PFS of 6.9 months and an ORR of 35.3%. Treatment was usually well tolerated, with the most common adverse events being thrombocytopenia and anemia. Critically, patients who needed dose reductions maintained similar efficacy rates to those without dose reductions, indicating that toxicity control did not affect treatment benefit.

Comparison with global data

The results can be placed into perspective with pivotal trials of innovator T-DM1 (Kadcyla®). The EMILIA trial showed a median PFS of 9.6 months and an OS of 30.9 months compared with lapatinib plus capecitabine [2], and the TH3RESA trial showed an OS benefit in highly pretreated patients [3]. The reduced PFS in our cohort is likely reflective of the real-world population, which had a heavy burden of comorbidities, prior exposure to multiple HER2-directed agents, and widespread metastatic disease. Such differences between trial and real-world outcomes are well recognized in oncology practice [6].

Real-world studies of innovator T-DM1 have shown a median PFS of 6.1 months and OS of 14.4 months, with better outcomes in hormone receptor-positive disease (PFS: 8.1 vs 4.1 months; p = 0.035) [11]. These findings align with our results, particularly the observed trend toward longer PFS in PR-positive patients.

Indian experience with biosimilars

Evidence from India on trastuzumab emtansine biosimilars remains limited. A multicenter randomized comparability trial confirmed pharmacokinetic, efficacy, and safety equivalence of Ujvira® to reference T-DM1 [5]. A single-center retrospective series (n = 30) reported a median PFS of 14 months and OS of 24 months, with manageable toxicity [12]. Other small series have shown PFS around six months and ORR up to 81% [13]. Compared with these reports, our shorter PFS likely reflects the broader and more comorbid nature of our population, with 30 patients (58.8%) diabetic and 31 (60.8%) hypertensive, along with heavier prior therapy exposure. These findings underscore the importance of real-world evidence in complementing trial data, especially in LMICs where patient characteristics often differ from global registrational studies.

Subgroup insights

**Figure 4:**
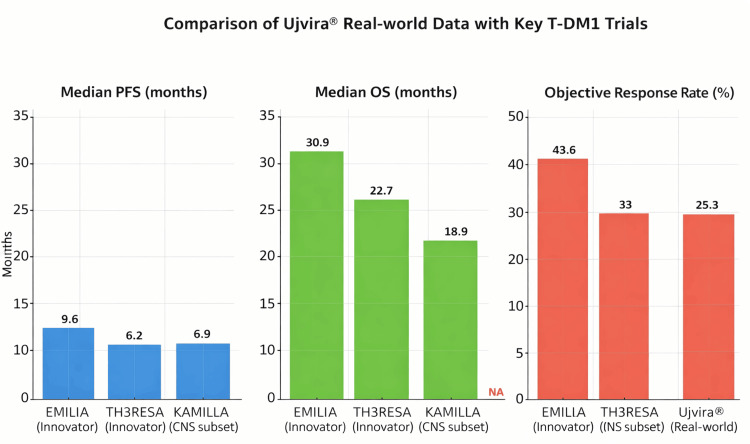
Comparison of various trials reported median PFS, OS (months) and objective response rate (%) with real world evidence data of Ujvira® (ZRC-3256; Zydus Lifesciences, India) PFS: progression-free survival, OS: overall survival.

Exploratory subgroup analyses in our study provide observations not previously reported in Indian biosimilar cohorts; however, these findings should be interpreted cautiously and considered hypothesis-generating due to limited statistical power. Brain metastases were associated with numerically worse outcomes (median PFS 6.3 months), consistent with the poor prognosis of CNS disease. This parallels findings from the KAMILLA trial, where innovator T-DM1 demonstrated a median PFS of 5.5 months in brain metastases [4]. Diabetes mellitus was associated with shorter PFS, suggesting it may be a negative prognostic factor. Given the high prevalence of metabolic comorbidities in Indian breast cancer patients, this warrants further study. PR positivity and FISH positivity were associated with numerically longer PFS. While not statistically significant, these patterns add potential prognostic value and may inform biomarker-driven stratification in future research. Dose reductions did not compromise efficacy, echoing findings from EMILIA and TH3RESA as depicted in Figure 4 [2,3].

Safety profile

The toxicity profile observed was consistent with both global and Indian data. Thrombocytopenia and anemia were the most common adverse events, with grade ≥3 toxicities being infrequent. This aligns with the randomized comparability trial [5] and retrospective Indian series [12,14]. Case reports have described durable benefit in selected patients, including those with brain metastases [7,15]; however, our study emphasizes the heterogeneity of real-world outcomes and the need for larger datasets to clarify subgroup effects.

Limitations

This study has several limitations. The retrospective, single-center design introduces potential selection and reporting bias. Moreover, the modest sample size limited statistical power, particularly for subgroup analyses, increasing the risk of type II error. In addition, OS data were underdeveloped at the time of analysis, limiting the ability to compare them with international survival benchmarks. Larger, prospective, multicenter real-world studies are warranted to validate these findings and improve the generalizability of outcomes across diverse patient populations. However, the strength of the study lies in its subgroup breakdown, focus on comorbidities, and realistic analysis of dose adjustments, which are not frequently addressed in the biosimilar literature.

## Conclusions

This real-world analysis confirms that Ujvira®, a trastuzumab emtansine biosimilar, provides clinical outcomes and tolerability consistent with the reference T-DM1 in Indian patients with HER2-positive metastatic breast cancer. Despite a highly pretreated and comorbid population, subgroups maintained PFS and response. Notably, dose changes did not affect efficacy, indicating the flexibility and safety of the treatment in clinical practice. Ujvira® can provide a practical and fair alternative for treating HER2-positive disease in LMICs due to its ability to extend access to cost-effective targeted therapy. Future studies with larger, multicenter cohorts and more mature overall survival data are needed to confirm these real-world findings and better define predictors of long-term benefit.

## Appendices

**Table 4:** Institutional practices for imaging, response assessment, and disease evaluation

Parameter	Institutional practice
Imaging modality	Contrast-enhanced computed tomography (CECT)
Anatomical coverage	Chest, abdomen, and pelvis
Baseline imaging	Performed within 4 weeks prior to treatment initiation
Follow-up imaging frequency	Every 8–12 weeks during treatment, or earlier if clinically indicated
Response assessment criteria	RECIST version 1.1
Assessment personnel	Institutional radiologists experienced in oncologic imaging
Target lesion selection	As per RECIST v1.1 guidelines at baseline
Definition of response	Complete response, partial response, stable disease, and progressive disease as per RECIST v1.1
Confirmation of progression	Radiological progression on follow-up imaging or unequivocal clinical progression
Imaging at treatment discontinuation	Performed at investigator discretion based on clinical status
